# SARS-CoV-2 infection of endothelial cells, dependent on flow-induced ACE2 expression, drives hypercytokinemia in a vascularized microphysiological system

**DOI:** 10.3389/fcvm.2024.1360364

**Published:** 2024-03-21

**Authors:** Christopher J. Hatch, Sebastian D. Piombo, Jennifer S. Fang, Johannes S. Gach, Makena L. Ewald, William K. Van Trigt, Brian G. Coon, Jay M. Tong, Donald N. Forthal, Christopher C. W. Hughes

**Affiliations:** ^1^ Department of Biomedical Engineering, University of California, Irvine, CA, United States; ^2^ Department of Pediatrics, School of Medicine, Institute for Clinical and Translational Science, University of California, Irvine, CA, United States; ^3^ Department of Molecular Biology and Biochemistry, University of California, Irvine, CA, United States; ^4^ Division of Infectious Diseases, School of Medicine, University of California, Irvine, CA, United States; ^5^ Cardiovascular Biology Research Program, Oklahoma Medical Research Foundation, Oklahoma City, OK, United States; ^6^ Department of Cell Biology, University of Oklahoma Health Sciences Center, Oklahoma City, OK, United States

**Keywords:** COVID-19, endothelial dysfunction, hypercytokinemia, microphysiological systems, shear stress

## Abstract

**Background:**

Severe acute respiratory syndrome coronavirus 2 (SARS-CoV-2), responsible for COVID-19, has caused nearly 7 million deaths worldwide. Severe cases are marked by an aggressive inflammatory response known as hypercytokinemia, contributing to endothelial damage. Although vaccination has reduced hospitalizations, hypercytokinemia persists in breakthrough infections, emphasizing the need for disease models mimicking this response. Using a 3D microphysiological system (MPS), we explored the vascular role in SARS-CoV-2-induced hypercytokinemia.

**Methods:**

The vascularized micro-organ (VMO) MPS, consisting of human-derived primary endothelial cells (ECs) and stromal cells within an extracellular matrix, was used to model SARS-CoV-2 infection. A non-replicative pseudotyped virus fused to GFP was employed, allowing visualization of viral entry into human ECs under physiologic flow conditions. Expression of ACE2, TMPRSS2, and AGTR1 was analyzed, and the impact of viral infection on ACE2 expression, vascular inflammation, and vascular morphology was assessed.

**Results:**

The VMO platform facilitated the study of COVID-19 vasculature infection, revealing that ACE2 expression increased significantly in direct response to shear stress, thereby enhancing susceptibility to infection by pseudotyped SARS-CoV-2. Infected ECs secreted pro-inflammatory cytokines, including IL-6 along with coagulation factors. Cytokines released by infected cells were able to activate downstream, non-infected EC, providing an amplification mechanism for inflammation and coagulopathy.

**Discussion:**

Our findings highlight the crucial role of vasculature in COVID-19 pathogenesis, emphasizing the significance of flow-induced ACE2 expression and subsequent inflammatory responses. The VMO provides a valuable tool for studying SARS-CoV-2 infection dynamics and evaluating potential therapeutics.

## 1 Introduction

The SARS-CoV-2, or COVID-19, pandemic has required major adaptations in how the medical and scientific community approaches infectious disease prevention and study (1, 2). One primary need has been the rapid development of new tools to analyze viral infection and the subsequent development of vaccines to combat the global dissemination of this deadly pathogen (3). To address the dearth of apt translational models for studying the pathogenesis of COVID-19, we have utilized a 3D microphysiological system platform—the vascularized micro-organ (VMO) model (4)—to interrogate the role of the vasculature in driving hypercytokinemia, which is a crucial pathological indicator associated with SARS-CoV-2 infection (5).

While COVID-19 was initially characterized as an infection that predominately manifests in the respiratory system, it is now also recognized to be a vascular disease (6), with potential long-term sequelae likely arising due to micro-clots associated with dysregulation of thrombosis in many patients (7). While the long-term effects of COVID-19 infection appear to be mediated by these thrombotic events, the most severe acute impact of the disease is driven by profound hypercytokinemia, a process associated with uncontrolled upregulation of key inflammatory mediators such as IL-1 and IL-6, and endothelial cell (EC) leukocyte adhesion molecules such as ICAM-1 and VCAM-1 (8, 9). Of note, however, is that IL-6 also drives the expression of pro-coagulation factors (10), potentially linking acute cytokine release with downstream coagulation.

This cascading inflammatory response frequently results in pneumonia and, in severe cases, acute respiratory distress syndrome, accounting for the high mortality prior to the introduction of the COVID-19 vaccine (11). Therapies such as glucocorticoids and antivirals such as remdesivir have been developed to combat these cases; however, the biological mechanisms that produce hypercytokinemia in COVID-19 patients remain elusive, although the use of tociluzumab, targeted to the IL-6 receptor, has proven useful in many patients (12). To further our understanding of the pathology of COVID-19 and the role of the vasculature, we have challenged the VMO platform with a non-replicative pseudotyped SARS-CoV-2 virus, allowing us to model and visualize viral entry into human EC in a system that closely recapitulates human vasculature. The VMO consists of human-derived primary EC and stromal cells suspended in a hydrogel matrix housed within a polydimethylsiloxane microfluidic system (13). Over the course of a few days, a complex vascular network forms that is then perfused by a blood substitute, which nourishes the surrounding tissue. These microvascular networks demonstrate key characteristics of human vasculature, such as tight junctions, expression of vascular markers, and response to inflammatory stimuli, and can support a variety of tissues, including heart, liver, pancreas, brain, and various tumors (4, 14, 15).

The key mediator of SARS-CoV-2 viral entry into host cells is angiotensin-converting enzyme 2 (ACE2), which acts as a receptor for the viral spike protein (16). While other renin-angiotensin system mediators, such as AGTR1, play a role in the resultant hypercytokinemia (17), ACE2 expression on the surface of EC is critical to the initial infection, and without adequate expression, viral infection fails to occur (18). The primary challenge of studying SARS-CoV-2 infection in commonly utilized models of the vasculature is the dependence of ACE2 expression on the shear force EC are exposed to *in vivo* (19, 20). As the VMO provides flow and shear force equivalent to that found in human capillary networks, the platform provides a unique model in which to study not only how ACE2 mediates viral infection and how viral entry clears ACE2 from the surface of the EC but also how these events trigger cytokine release from the EC lining the vascular network.

## 2 Materials and methods

### 2.1 Microfluidic device design

**Figure 1:**
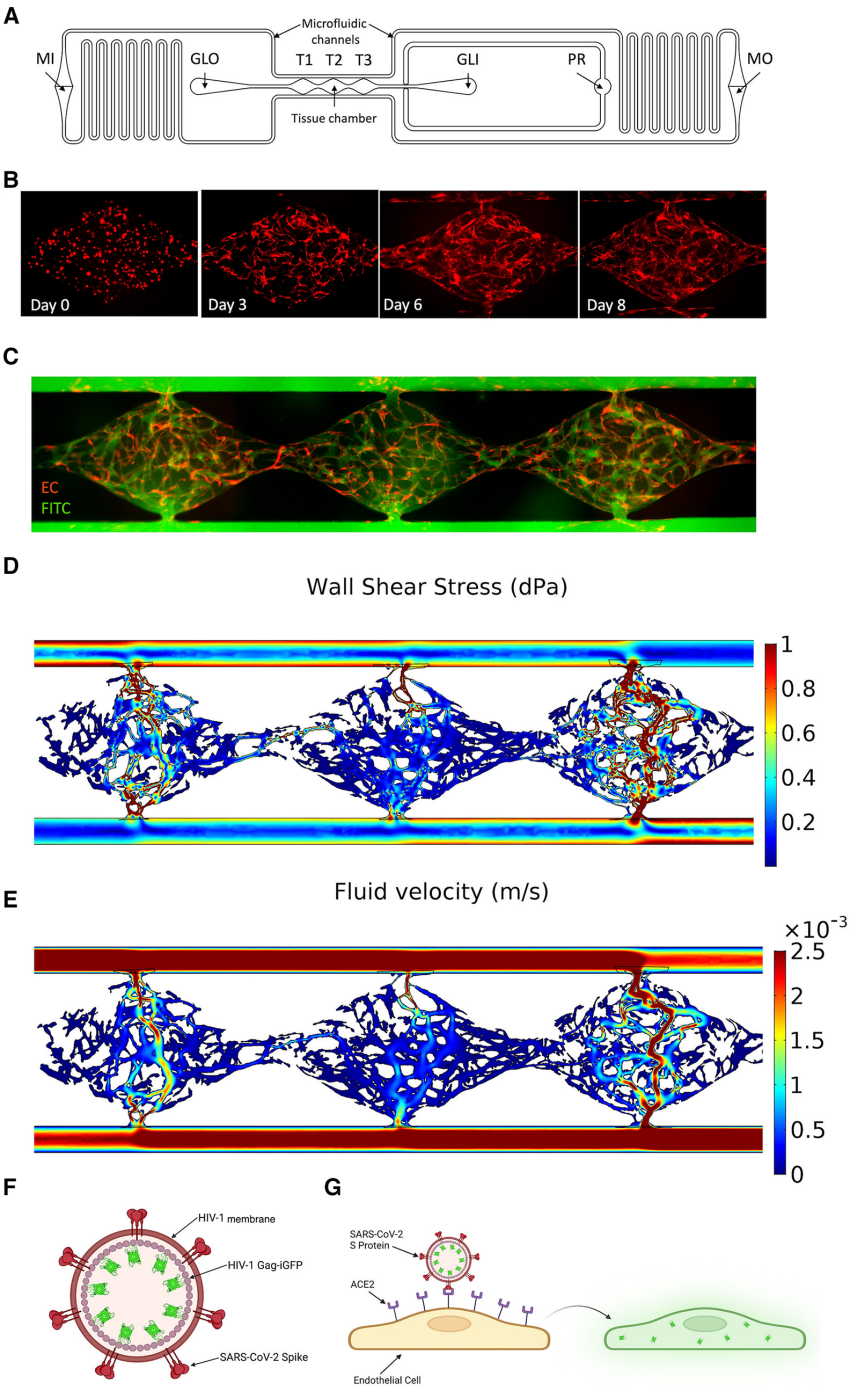
Development of a vascularized micro-organ microphysiological system and SARS-CoV-2 pseudotyped virus. (**A**) Schematic of an individual microfluidic device unit. Hydrostatic pressure drives medium from the inlet (MI) through the microfluidic channels and tissue chamber (T1–T3) to the medium outlet (MO). Hydrogel is loaded via the gel loading inlet (GLI) and outlet (GLO) with the pressure regulator (PR) to prevent leak into the microfluidic channel in the case of over-pressure during injection. (**B**) Development of the vasculature over time. EC labeled red; fibroblasts unlabeled. (**C**) Perfusion of 70 kDa FITC-Dextran through the vasculature (EC eRFP) at day 8. (**D**) COMSOL modeling of the shear stress (dynes/cm^2^) through each tissue chamber. (**E**) Quantification of fluid flow through each chamber (m/s). (**F**) Schematic of SARS-CoV-2 pseudotyped virus. SARS-CoV-2 spike protein is inserted into the membrane of an HIV-1 envelope with a Gag-iGFP construct. (**G**) Schematic showing infectivity of the pseudotyped virus into EC using ACE2, leading to GFP expression.

The design of the platform is a modified version of our previously published VMO microphysiological system (13, 14). The platform is designed to fit a standard bottomless 96-well plate (FLUOTRAC^TM^, Greiner Bio-One). The design is shown in Figure 1A and comprises two polydimethylsiloxane (PDMS) layers adhered to a commercial 96-well plate with the wells aligned with the microfluidic features. The middle layer contains 16 microfluidic device units within the PDMS device layer, and the bottom layer is a thin transparent polymer membrane (Rogers Corp, HT-6240).

A single microfluidic device unit covers six horizontal wells of the 96-well plate, with the tissue chamber containing three diamond-shaped units (T1–T3) covering one well. A gel loading inlet (GLI) and outlet (GLO) are connected to either side of the tissue chamber, and each is aligned with an additional well. Each diamond-shaped unit is 2 mm in length and 1 mm in width, connected to 200 μm wide microfluidic channels through anastomosis points. The entire design is 200 μm thick. A redundant gel outlet is integrated into the GLI to act as a pressure regulator (PR) and prevent hydrogel from entering the microfluidic channel when the loading pressure exceeds the anastomosis point burst valve pressure. The microfluidic channels contain resistors in an asymmetric design to generate a hydrostatic pressure drop across the tissue chamber. This drives interstitial flow as the medium moves from the medium inlet (MI) to the medium outlet (MO).

### 2.2 Microfluidic device fabrication

The fabrication of the VMO has been previously described (4, 14). Briefly, a customized polyurethane (PU) master mold is fabricated from a two-part PU liquid plastic (Smooth-Cast 310, Smooth-On Inc.). A PDMS replica is made from the master mold, and holes are punched on the replica for the inlets and outlets (MI, MO, GLI, GLO, PR). The replica is secured to the bottom of a 96-well plate by chemical glue and oxygen plasma treatment. The bottom thin transparent membrane is bonded to the PDMS layer with oxygen plasma treatment. The complete platform is placed in a 60°C oven overnight. Before the platform is loaded with cells, a standard 96 well-plate polystyrene lid with condensation rings (Greiner Bio-One) is placed on top, and both parts are sterilized using a UV light for 30 min.

### 2.3 Cell culture

Human endothelial colony-forming cell-derived endothelial cells (ECFC-EC) were isolated from cord blood (21), expanded on 0.1% gelatin-coated flasks, and cultured in EGM-2 (Lonza). ECFC-EC were transduced with lentiviruses encoding azurite fluorescent protein (Azurite/Addgene) and used between passages 7–9. Note that images are false-colored so that ECs appear red. Normal human lung fibroblasts (NHLF) (Lonza) were cultured in DMEM (Corning) containing 10% FBS (VWR) and used between passages 7–10. All cells were cultured at 37°C/20% O_2_/5% CO_2_ and checked for mycoplasma contamination using MycoAlert (Lonza) before use.

### 2.4 Microfluidic device loading

The tissue chamber was loaded with ECFC-EC and NHLF. The cells were trypsinized, lifted, and resuspended at a 1.4:1 ratio at a density of 1.4 × 10^7^ cells/ml in an 8 mg/ml fibrinogen solution (Sigma-Aldrich). Next, the cell-matrix solution was mixed with 3 U/ml thrombin (Sigma-Aldrich) and loaded in the tissue chambers. The VMO was placed into a 37°C incubator for 15 min to enable polymerization of the cell-matrix mix. Next, laminin (1 mg/ml, Life Technologies) was pushed through the microfluidic channels and incubated for 15 min at room temperature. Finally, EGM-2 culture medium was pushed through the microfluidic channels and dispensed in the inlet and outlet wells to generate hydrostatic pressure heads that drive perfusion. The medium was changed every day to maintain the pressure heads.

### 2.5 Fluorescence imaging and analyses

Fluorescence images were captured on an Olympus IX70 inverted microscope using SPOT software (SPOT Imaging), a Nikon Ti-E Eclipse epifluorescent microscope with a 4xPlan Apochromat Lambda objective, and an Agilent BioTek Lionheart FX microscope. In addition, confocal images were captured on a Leica TCS SP8 confocal microscope using a standard 10× air or 20× multi-immersion objective (Leica Microsystems).

FIJI (22) was used to determine each microfluidic unit's mean fluorescent intensity (MFI). Before measuring the average pixel intensity, all images underwent thresholding and default background subtraction with the same setting using a custom macro. Then, all values were normalized to the mean control fluorescent intensity for analysis.

### 2.6 Perfusion testing

To test the vascular leak of device networks, 70 kDa FITC-dextran (Sigma-Aldrich) was diluted in EGM-2 to 50 μg/ml and added to well MI for perfusion through the device. Images were taken after 15 min, and the vessel patency was assessed by demonstrating dextran flow through the entirety of the vascular chamber with no leakage at any of the anastomosis points.

### 2.7 Generation of pseudotyped SARS-CoV-2 virus and infection of the microfluidic device

Pseudotyped SARS-CoV-2 virions were generated by co-transfecting 1.5 × 10^7^ 293-*T* cells with a single round infectious HIV-1 NL4-3 Gag-iGFP *Δ*Env plasmid as well as a SARS-CoV-2 spike protein expressing plasmid [pcDNA 3.1 SARS CoV-2 S or pcDNA3.3_SARS2_omicron BA.2 (23)]. Plasmids (1 µg plasmid per 1 × 10^6^ cells) were mixed with polyethyleneimine (PEI) at a DNA/PEI ratio of 1:3 and added to the cells. After 3–4 days, cell supernatants were harvested and cleared from cells. Virus aliquots were stored at −80°C. To concentrate the virus, frozen supernatants were thawed and spun (14,000 rpm) in a microcentrifuge for 75 min at 4°C, and viral pellets were resuspended in the respective medium. Virus infectivity was determined by infecting 1 × 10^4^ human ACE2 receptor expressing HEK 293 T target cells (BEI Resources) per well with a serial dilution of the CoV-2/HIV-1 pseudotyped virus particles. After 2–3 days, cells were detached, washed, and fixed with 4% PFA. Target cells were subsequently analyzed by flow cytometry (NovoCyte flow cytometer; ACEA) for green fluorescence protein expression. After performing perfusion tests to confirm the robustness of the vascular networks, the pseudotyped virus was added to the device inlet. Unless specified, 7 × 10^5^ IFU/ml of pseudotyped virus were used. Recombinant angiotensin II (rAngII, Sigma-Aldrich, 4474-91-3) was added to the inlet at a 300 pM concentration to activate the renin-angiotensin system. After 24 h, medium heights were reestablished by taking the media from the outlet well and placing it back in the inlet. After 48 h, the effluent was collected.

### 2.8 Immunostaining

VMO devices were fixed overnight at 4°C by replacing the circulating media in the inlet and outlet reservoirs with 4% paraformaldehyde diluted in phosphate-buffered saline (PBS). Following fixation, the transparent polymer seal was removed, exposing VMO networks embedded within the microfluidic feature layer, which was then placed face-up in each well of either a 4-well chamber slide (Nunc) or a 24-well plate. Fixed devices were then post-fixed for an additional 20 min in 4% paraformaldehyde at room temperature and then incubated in blocking solution [PBS containing 3% bovine serum albumin (BSA) and 0.1% Triton X, supplemented with 1% donkey serum] for 1 h at room temperature. Devices were then incubated in primary antibody diluted in staining solution [PBS containing 1% bovine serum albumin (BSA) 0.1% Triton X] overnight at 4°C. Following the wash, devices were incubated in fluorescently conjugated secondary antibodies diluted in staining solution for 2 h at room temperature. This was followed by incubation in 10 ug/ml Hoechst 33,342 (Sigma 14,533) for 15 min at room temperature. Devices were then washed in PBS and mounted in 4-well glass chamber slides (Nunc Lab-Tek, Thermo 177,399) in Vectashield (Vector Labs H1300) anti-fade mounting media. High-magnification multi-channel images of stained VMO devices were acquired using a Leica SP8 confocal microscope. Primary antibodies used: anti-ACE2 (Novus NBP2-67692, 1:300), anti-TMPRSS2 (Abcam ab242384, 1:300), anti-CD31 (Abcam ab28364, 1:300). Fluorescently conjugated secondary antibodies used: anti-Rabbit-Alexa 488 (1:500), anti-mouse-Alexa 568 (1:500), anti-goat-Alexa 647 (1:500).

### 2.9 ELISAs

Effluent from devices was collected after 48 h of exposure to experimental treatments. If not used immediately, the effluent was stored in the vapor phase of liquid nitrogen. Before use, the effluent was allowed to reach room temperature and was centrifuged at 2000g for 10 min. ELISAs for IL-6 (Abcam, ab178013), sICAM-1 (Abcam, ab229383), sVCAM-1 (Abcam, ab223591), Factor VIII (Abcam, ab272771), and IL-1β (Thermo Fischer Sci, BMS224-2) were run. A 100 K molecular weight cutoff protein concentrator (Thermo Scientific, 88523) was used to pool two samples and concentrate them for the Factor VIII ELISA. The drc package (24) in RStudio (25) fit the standard data to point curves as the manufacturers’ protocols recommended. Values that fell outside the range of the standards were excluded from the analysis.

### 2.10 RNA isolation and qRT-PCR analysis

For RNA isolation, the protective plastic cover underneath the platform was removed. A razor blade was used to extract the tissue chamber and surrounding chamber inlets and outlets. Carefully, tweezers were used to separate the bottom polymer layer from the PDMS layer. Approximately 20 μl of RNA lysis buffer from the Quick RNA micro prep kit (Zymo Research, R1051) was added dropwise to the tissue chamber and bottom polymer layer and allowed to sit for 3–5 min. RNA lysis buffer was collected and diluted in a 1:1 ratio with pure ethanol before transfer to a Zymo-Spin IC Column. The RNA was then isolated following the manufacturer's protocol. The RNA was checked for quantity and quality using a NanoDrop before storage at −80°C or immediate conversion into cDNA.

Total purified RNA was synthesized into cDNA with the iScript cDNA Synthesis Kit (BioRad, 1708891) before use in quantitative real-time polymerized chain reaction (qRT-PCR) (BioRad). The average cycle threshold values were normalized using 18S expression levels and compared to their appropriate controls. Any cycle threshold values greater than 40 had a fold change set to one. All samples were measured in triplicate. Primers were designed with PrimerQuest Tool and synthesized by Integrated DNA Technologies.

### 2.11 Ibidi chip

ECFC-EC were plated into the 0.4 mm *μ*-Slide I Luer (Ibidi, 80176) at 1 × 10^6^ cell/ml and 100 μl and allowed to adhere overnight following the manufacturer's protocol. Next, the cells were exposed to 4 h of shear stress at 0.5, 1.0, or 2.0 dynes/cm^2^ using a syringe pump (Pump Systems Inc) with the flow rate set via ibidi's recommended values. After 4 h, the chips were washed with DPBS 3X, and RNA lysis buffer using the Quick RNA micro prep kit (Zymo Research, R1051) was pushed through the chamber following the manufacturer's protocol. As previously described, steps were followed to isolate the RNA and run qPCR.

### 2.12 Vessel morphometry

Vascular network images were analyzed with AngioTool software (National Cancer Institute) (26) to quantify vessel area, length, lacunarity, and the number of vascular junctions and endpoints. The mean vessel diameters were computed using a modified version of the REAVER package (27) to output all vessel diameters instead of the mean for the entire network. The forked repository is available on GitHub (https://github.com/cjhatch/public_REAVER_diams). For vessel morphometry, replicates from two loadings were used. In loading 1, the control condition had biological replicates from 7 tissue chambers, with 21 diamond-shaped units quantified, while the pseudovirus and rAngII condition had 7 tissue chambers, with 16 diamond-shaped units quantified. For loading 2, the control condition had 4 tissue chambers, with 9 diamond-shaped units quantified, while the pseudovirus and rAngII condition had 7 tissue chambers, with 21 diamond-shaped units quantified.

### 2.13 NFκB reporter and monolayer response

ECFC-EC were transduced with an NF*κ*B reporter and plated into 12-well plates. In brief, a Generation II lentiviral construct consisting of three tandem NF*κ*B response elements upstream of the mCherry coding sequence, followed by a PGK reporter upstream of a SNAP-tag constitutive reporter were packaged into lentivirus and used to infect ECFC-EC. Thus, mCherry expression was driven by NF*κ*B expression, which allowed for indirect quantification of inflammatory status via real-time fluoroscopy. Following validation of the reporter construct, 1.5 ml of effluent was collected from the VMOs and then layered onto NF*κ*B reporter-transduced EC. Each well was imaged at 0, 24, and 48 h. MFI was calculated from each well to quantify the inflammatory response to downstream mediators.

### 2.14 Drug treatments

After performing perfusion tests to confirm the robustness of the vascular networks, pharmacological agents were added at the same time as the pseudotyped virus to test their ability to limit infectivity. Recombinant ACE2 (25 μg/ml, Sigma-Aldrich, SAE0065-50UG) or camostat mesylate (100 μM, Sigma-Aldrich, SML0057-10MG) was premixed with medium ± pseudotyped virus ± rAngII for 30 min before perfusing through devices. The pressure heads were readjusted by recycling media from the outlet well back into the inlet after 24 h, and effluent was collected after 48 h. Images were taken at 0, 24, and 48 h.

### 2.15 Computational fluid dynamics

Image masks were converted into .tiff RGB files using FIJI. The image files were then binarized, skeletonized, and traced using a custom MATLAB script. The traced images were converted to .DXF files using the DXFLib package (28) and imported into AutoCAD to overlay the schematics for the microfluidic devices. The traced vessels and microfluidic devices were imported into COMSOL 5.2.1. The velocity and shear stress were calculated using the laminar flow steady-state model, with water as the fluid material. Using the Bernoulli equation, all pressure heads were calculated based on medium height in the inlet/outlet wells to provide input to the COMSOL model.

### 2.16 Plotting and statistical analysis

All plots were generated in RStudio (25) using R version 4.0.3 with the ggplot2 package (29), ggpubr package (30), and gridExtra package (31).

**Figure 2:**
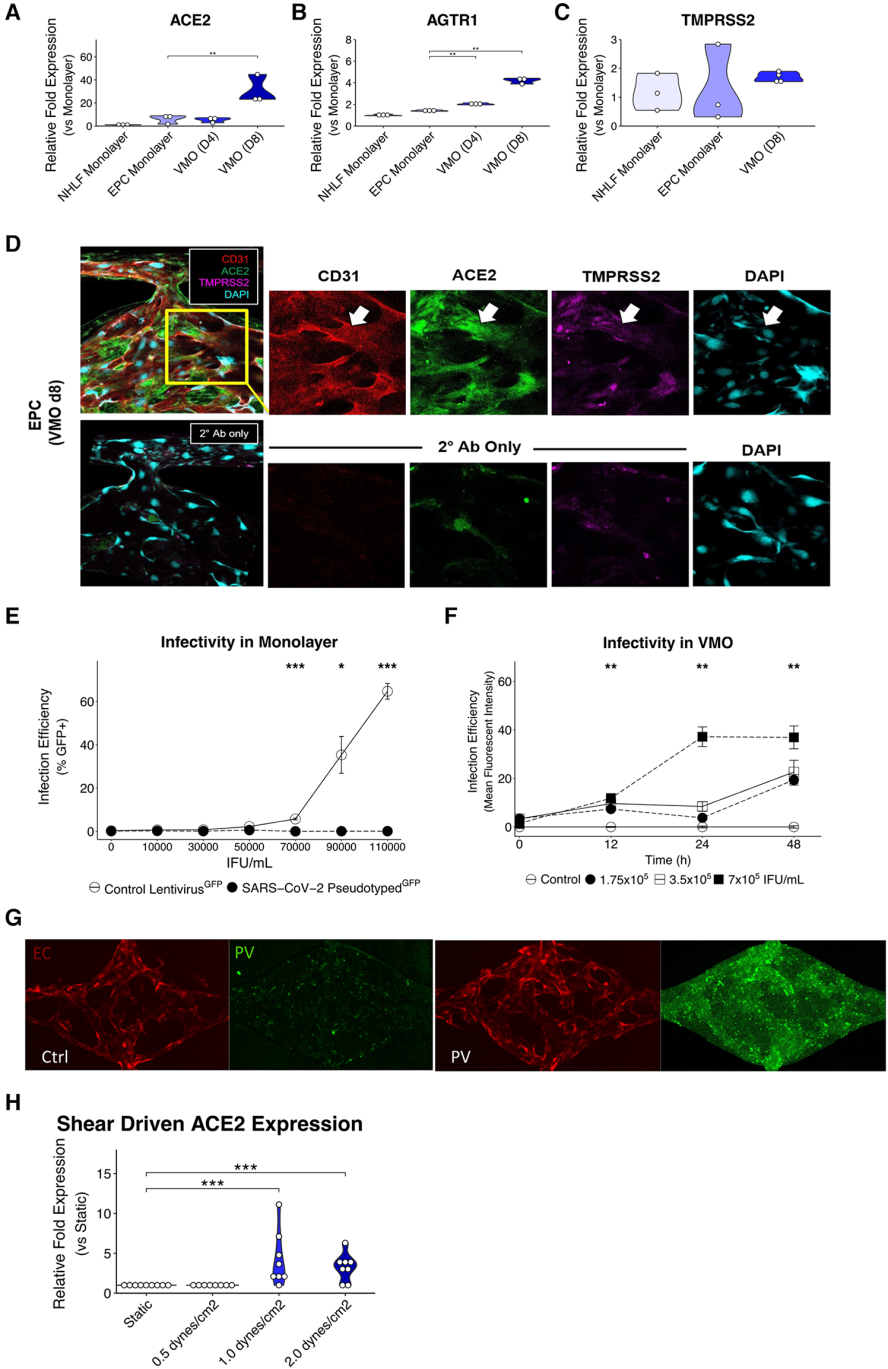
Upregulated ACE2 expression in the vascularized micro-organ supports SARS-CoV-2 pseudotyped infectivity. qPCR analysis of (**A**) ACE2, (**B**) AGTR1, and (**C**) TMPRSS2, which are necessary for SARS-CoV-2 infectivity, comparing monolayer normal human lung fibroblasts (NHLF), monolayer EC, the vascularized micro-organ (VMO) before (D4) and after (D8) the formation of perfusable vasculature. (**D**) Immunofluorescence staining of a D8 VMO showing CD31/PECAM1 (red), DAPI (blue), and localization of ACE2 (green) and TMPRSS2 (purple) to the endothelium. Showing nonspecificity of secondary antibodies alone. (**E**) Infectivity efficiency (mean fluorescent intensity) of SARS-CoV-2 pseudotyped virus compared to a lentivirus expressing green fluorescent protein (GFP). (**F**) Titering of GFP SARS-CoV-2 pseudotyped virus in a perfused VMO. Infectivity is measured as an increase in the mean fluorescent intensity of the GFP channel. (**G**) Subset of a D8 VMO EC (mCherry) and background fluorescence or fluorescence induced by infection with SARS-CoV-2 pseudotyped virus (GFP). Ctrl, control; PV, pseudotyped virus. (**H**) qPCR analysis of ACE2 expression induced by shear stress in an ibidi microfluidic chip. *<0.05, **<0.01, ***<0.001.

For ELISAs and qPCR data, one-way ANOVAs with posthoc Tukey's HSD tests were run using the rstatix package (32) for parametric data. For nonparametric data, a Kruskal–Wallis H test was run with a Conover-Iman *post hoc* test using the conover.test package (33). For the time course for MFI infectivity at various doses (Figure 2F), an unbalanced two-way ANOVA on rank-transformed data was run using the car package (34), and the type III sum of squares was used to determine the significance of the main effects and interactions. Tukey's HSD was run to determine the significance between treatments.

## 3 Results

### 3.1 A microphysiological model for vascular SARS-CoV-2 infection

The VMO is a 3D vascularized microtissue platform comprising ECs and stromal cells (fibroblasts, pericytes, etc.) suspended in an extracellular matrix (ECM) that we have used as a base platform for the development of multiple tissue-specific models including heart, pancreas, liver, brain, and tumors. Here, we have used a modified version of this device to study infection of the vasculature by the COVID-19-causative agent, SARS-CoV-2 (Figure 1). We array 16 individual tissues in a 96-well plate format, where each unit consists of three interconnected tissue chambers that rely on hydrostatic pressure to drive interstitial flow through the vessels (Figure 1A). Once seeded as a dispersed cell suspension in gel the ECs and fibroblasts undergo vasculogenesis and angiogenesis over the course of 5–6 days to form a perfusable vascular network (Figures 1B,C) (13). ECs also migrate into the microfluidic channels that represent the artery and vein, and these anastomose with the vascular network to allow pressure-driven fluid flow from the artery, through the network, and out of the vein. The perfused vasculature thereby experienced physiologically-relevant fluid flow and shear stress (35) (Figures 1D,E).

### 3.2 Vasculature in the VMO is a target for SARS-CoV-2 infection

A non-replicative pseudotyped virus was generated, consisting of HIV-1 Gag fused to GFP and enveloped with either the original SARS-CoV-2 spike protein strain or the omicron strain (Figures 1F,G). The spike protein of COVID-19 recognizes the cell surface receptor ACE2 and is reliant on binding to it to enable membrane fusion (36), with TMPRSS2 acting to prime the spike protein (37). In healthy vasculature, angiotensin II, generated by ACE, is usually rapidly degraded by ACE2, limiting its effective range and duration of action. Downstream of ACE and ACE2 is AGTR1/AT1R, which responds to angiotensin II to activate the classical, pro-inflammatory arm of the renin-angiotensin system (38). We first compared the RNA expression levels of ACE2, TMPRSS2, and AGTR1 in endothelial monolayers and in the VMO on days 4 and 8 (Figures 2A–C). We observed a significant increase in ACE2 and AGTR1 expression in the VMO vasculature compared to cells in the monolayer, and expression was higher still once flow was fully established (day 8) compared to the early stages of lumen formation (day 4). There was no apparent change in TMPRSS2 mRNA expression in the VMO. We performed immunofluorescence staining and confirmed ACE2 and TMPRSS2 in the VMO (Figure 2D). Consistent with our expression data showing an extremely low level of ACE2 expression by monolayer EC; these cells were not infectable with pseudotyped virus; however, they were infectable with a control, VSV coat protein, GFP-expressing virus (Figure 2E). In contrast, the SARS-CoV2-pseudotyped virus readily infected the endothelium when perfused through the VMO for 48 h (Figures 2F,G), again consistent with our expression data showing induction of ACE2 under flow conditions. We hypothesized that the increase in ACE2 in the VMO is likely caused by exposure to shear stress, as demonstrated by others (19). To test this directly, we exposed monolayer ECs to a range of shear stresses comparable to those found at various points throughout the vascular network in the VMO. Analysis of mRNA expression revealed that ACE2 is indeed induced dose-dependently by flow (Figure 2H).

### 3.3 SARS-CoV-2-pseudotyped virus infection in the VMO generates local EC inflammation

Having demonstrated that ECs in the VMO are infectable by a SARS-CoV-2-pseudotyped virus, we next wished to determine the impact of infectivity on ACE2 mRNA expression levels and downstream inflammation, as others have shown decreasing mRNA ACE2 expression during the course of infection in patients and the impact it has on inflammation (39). As noted above, in the body, the effects of angiotensin (AngII), mediated by AGTR1, are transient due to the rapid degradation of AngII by ACE2. Peptide degradation products of this reaction (specifically Ang_1−7_) bind to the Mas receptor (MasR), which mediates an anti-inflammatory signal. Loss of ACE2 leads to prolonged activation of AGTR1 and loss of MasR signaling, the combination of which has been shown to be pro-inflammatory (40). We therefore tested whether recombinant AngII (rAngII) in the presence or absence of pseudotyped virus would lead to a pro-inflammatory EC phenotype in the VMO and potentially influence ACE2 expression. Consistent with our hypothesis, both rAngII and the pseudotyped virus reduced ACE2 mRNA expression levels in EC in the VMO (Supplementary Figures S1A,B). AGTR1 expression levels were also reduced.

**Figure 3:**
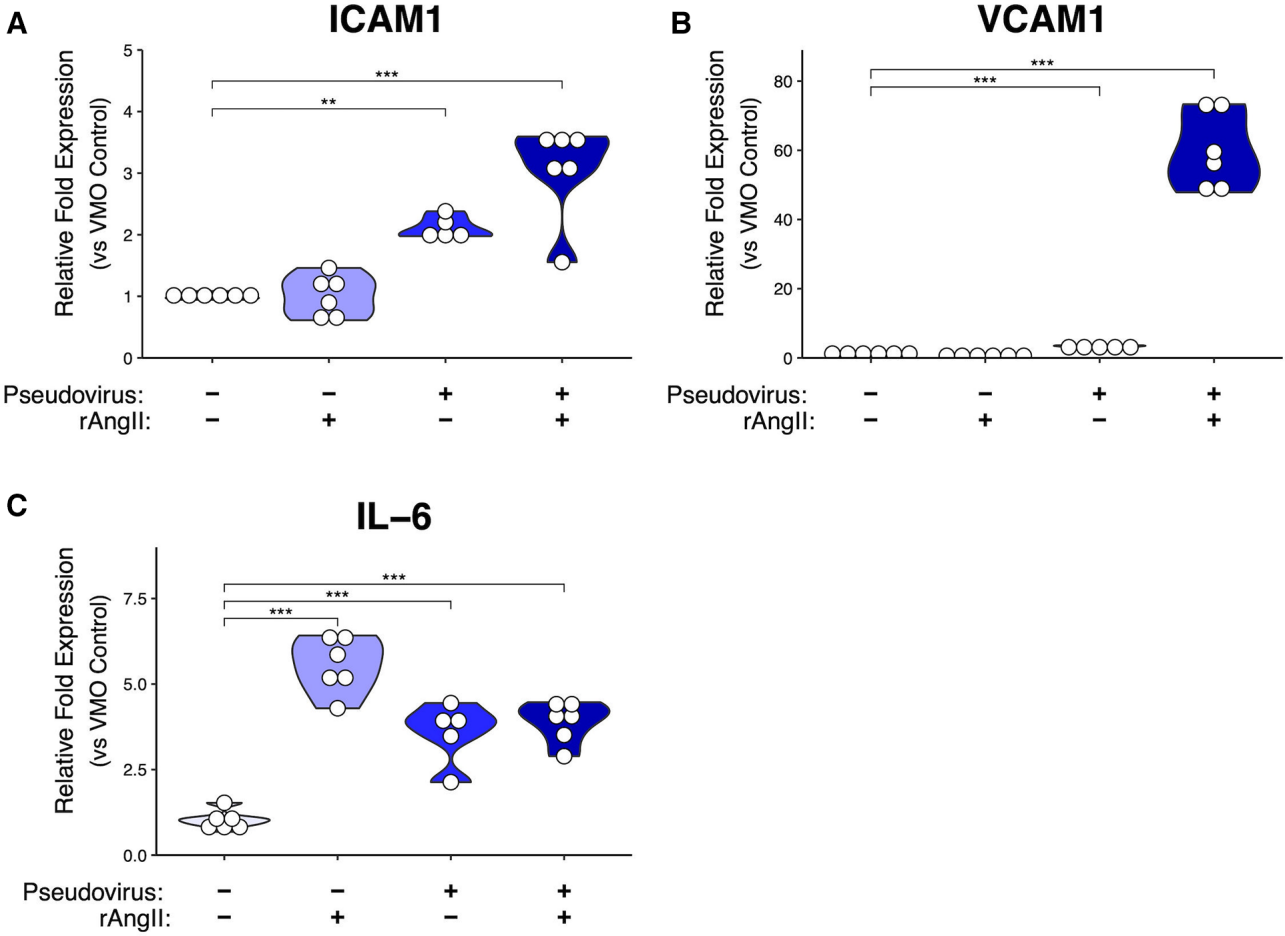
Modeled SARS-CoV-2 infection alters VMO transcriptomic profiles. In the VMO, qPCR analysis of pro-inflammatory markers (**A**) ICAM-1, (**B**) VCAM-1, and (**C**) IL-6 for perfused vascularized micro-organ (VMO) that have been treated with SARS-CoV-2 pseudotyped virus (pseudovirus), recombinant angiotensin II (rAngII) or both for 48 h. *<0.05, **<0.01, ***<0.001.

We then evaluated the expression of two key leukocyte adhesion molecules expressed by EC—ICAM-1 and VCAM-1—which are typically upregulated on EC in response to inflammatory stimuli such as IL-1 and TNF (41, 42), but also by the pleiotropic pro-inflammatory cytokine IL-6, the elevated expression of which is often associated with cytokine storms (43). We found both ICAM-1 and VCAM-1 genes were strongly upregulated by a combination of pseudotyped virus and rAngII (Figures 3A,B). Interestingly, ICAM-1 was also upregulated quite substantially by pseudotyped virus alone, whereas VCAM-1 was only marginally induced by this treatment, suggesting perhaps a higher required threshold for induction. IL-6 was induced by rAngII or virus alone, and there was no further induction in our system in combination (Figure 3C). Thus, our data suggest that downregulation of ACE2 mRNA expression levels as a consequence of viral infection in COVID-19 can lead to the expression of pro-inflammatory genes by the vasculature.

**Figure 4:**
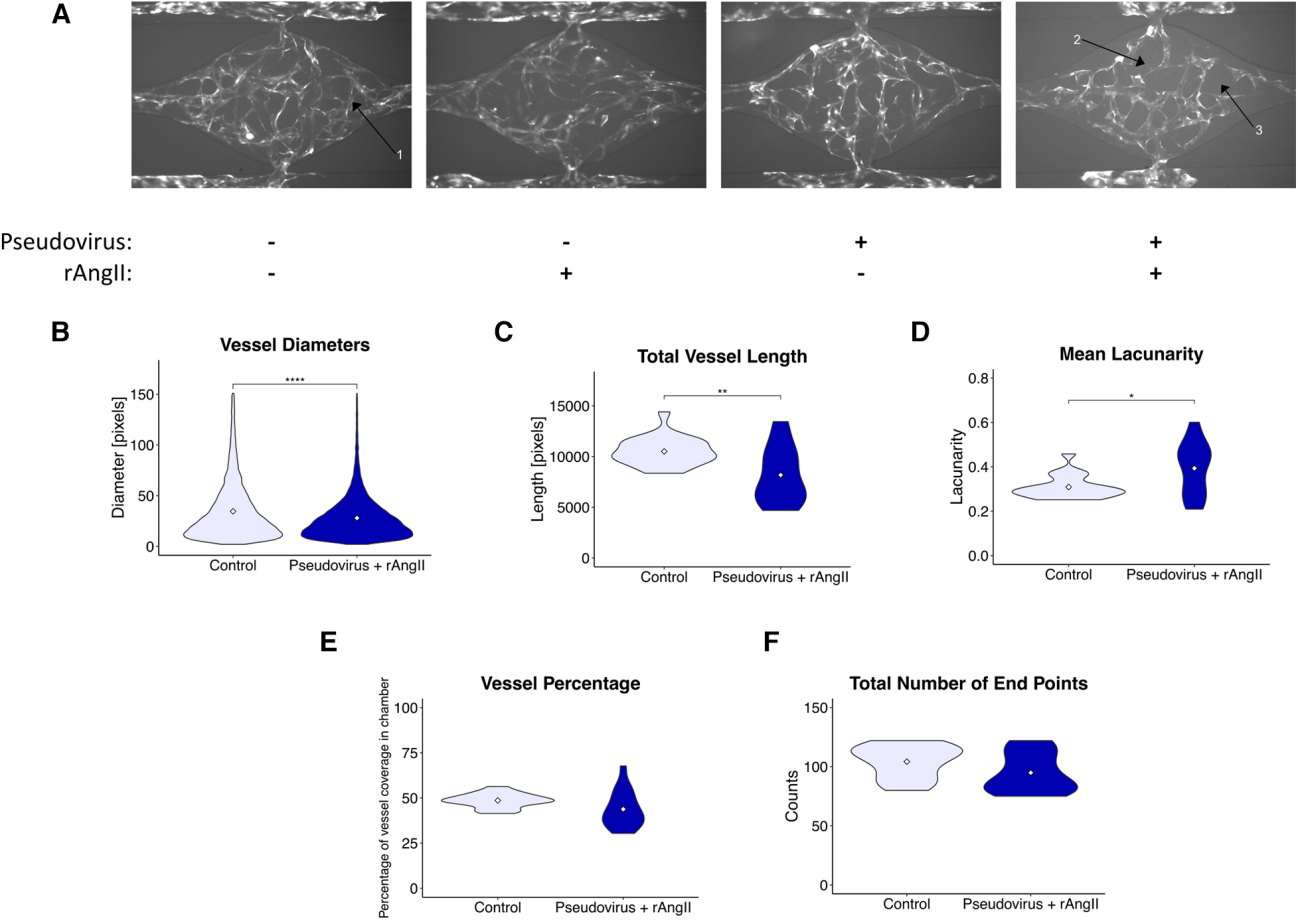
Modeled SARS-CoV-2 infection alters vessel morphometry. (**A**) Representative images of perfused VMOs treated with SARS-CoV-2 pseudotyped virus (pseudovirus) and/or recombinant angiotensin II (rAngII) for 48 h. Arrow 1: Under control conditions, vasculature is continuous and extends to the edges of the tissue chamber. Arrow 2: Discontinuous vessels, likely undergoing pruning and retraction. Arrow 3: Larger regions of perivascular space emerge due to vessel pruning. (**B–G**) Quantification of morphometry of devices showing changes in (**B**) vessel diameter, (**C**) total vessel length, (**D**) mean lacunarity, (**E**) vessel coverage percentage, and (**F**) total number of end points. *<0.05, **<0.01, ***<0.001, ****<0.0001. Control *n* = 11, Pseudovirus + rAngII *n* = 14 (See methods for more detail about replicates).

To better understand how this pro-inflammatory signature might disrupt the local vascular niche, we quantified morphological changes in the vasculature induced by the modeled COVID-19 infection (Figure 4A). After infection, we noted a modest reduction in vessel diameter (Figure 4B), total vessel length (Figure 4C), and an increase in lacunarity (Figure 4D), which characterizes vessel non-uniformity and is often increased in pathological vasculature (26). In addition, although not significant, there was a trend towards a reduction in vessel percentage coverage (Figure 4E); however, there was no change in the total number of endpoints (Figure 4F), suggesting that angiogenic sprouting was not induced.

### 3.4 SARS-CoV-2-pseudotyped virus infection in the VMO stimulates release of soluble inflammatory mediators

**Figure 5:**
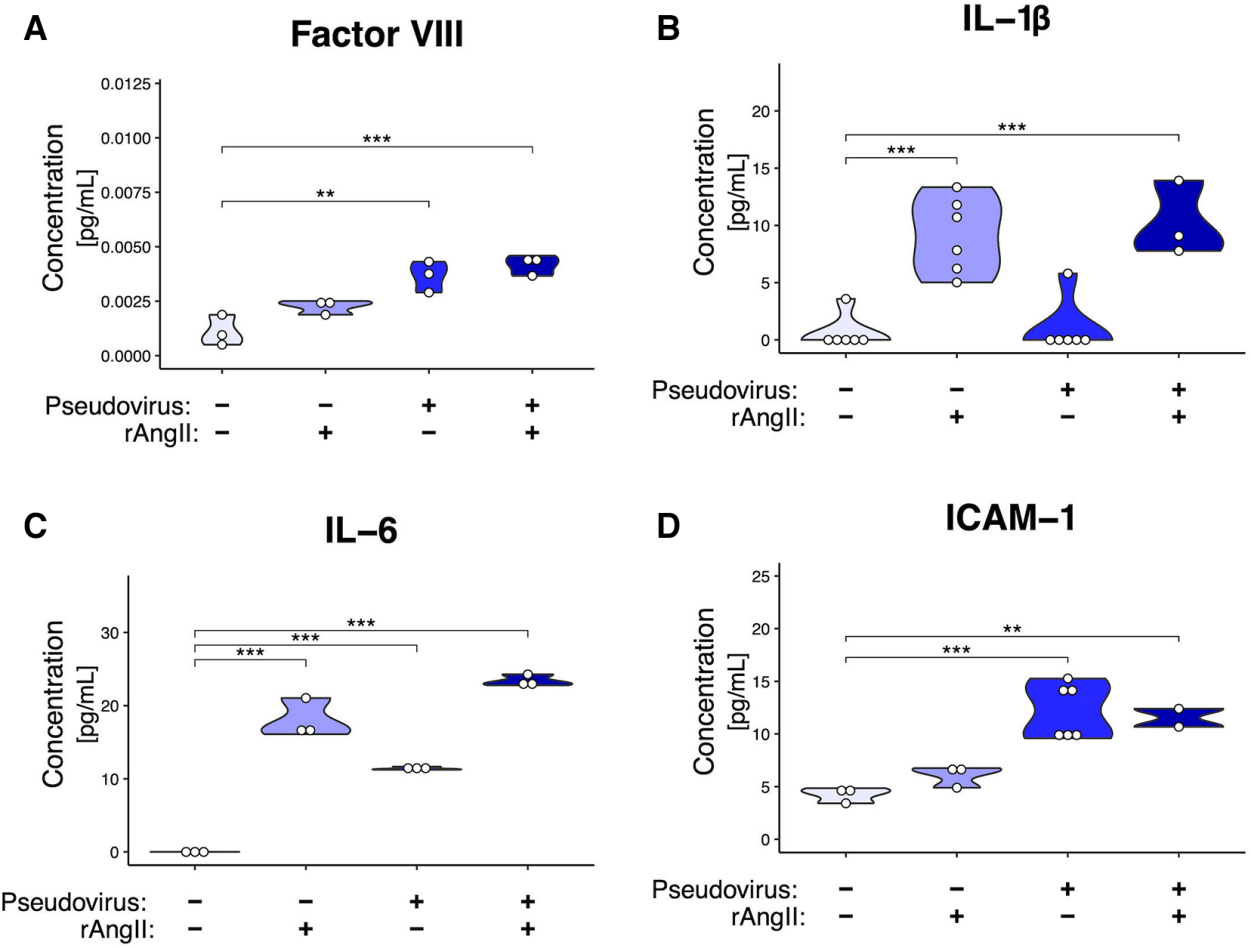
Modeled SARS-CoV-2 infection induces cytokine production. ELISAs from effluent collected from VMOs treated with SARS-CoV-2 pseudotyped virus (pseudovirus) and/or recombinant angiotensin II (rAngII) for 48 h. (**A**) Factor VIII, (**B**) IL-1β, (**C**) IL-6, (**D**) sICAM-1 protein content (pg/mL). *<0.05, **<0.01, ***<0.001.

To characterize the extent to which a localized COVID-19 infection could generate the pro-inflammatory and pro-thrombotic environment produced by infected ECs (44), we investigated inflammatory protein secretion via ELISA (Figure 5). Coagulation Factor VIII is secreted by EC Weibel-Palade bodies and is a cofactor for activating factor IX, which induces the intrinsic coagulation cascade (45). In perfused VMOs treated with rAngII, pseudotyped virus, or a combination of the two, there was an increase in coagulation Factor VIII secretion (Figure 5A). Additionally, there was increased secretion of several EC-derived inflammatory molecules previously identified in COVID-19 patients (46), including IL-1β, IL-6, and sICAM-1 (Figures 5B-D).

### 3.5 Pharmacological intervention blocks SARS-CoV-2-pseudotyped virus infection and cytokine release

**Figure 6:**
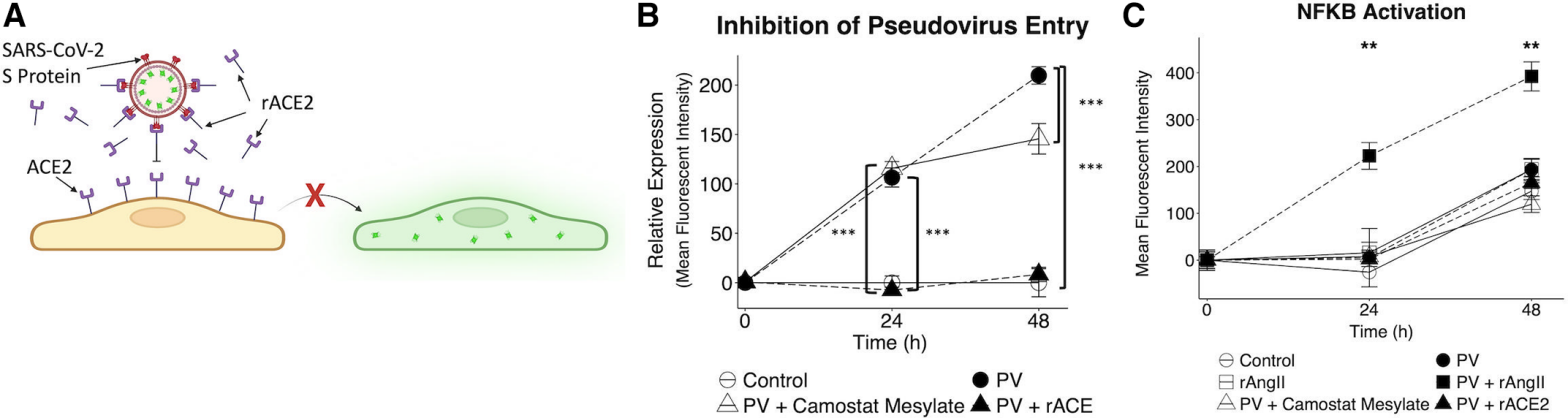
Pharmacological intervention inhibits SARS-CoV-2 pseudotyped virus entry and reduces downstream nF*κ*B activation. (**A**) Recombinant ACE2 (rACE2) prevented pseudotyped infectivity through competitive binding of the SARS-CoV-2 spike protein. (**B**) VMOs were treated with SARS-CoV-2 pseudotyped virus and rACE2 or camostat mesylate (camostat). The mean fluorescent intensity of the GFP pseudotyped virus was measured. Camostat mesylate had minimal protective ability, and rACE2 prevented infection. (**C**) Effluent from non-treated and treated devices was placed on EC transduced with an NF*κ*B induced mCherry reporter. The mean fluorescent intensity of mCherry was measured. *<0.05, **<0.01, ***<0.001.

Since the VMO can also be used to conduct drug studies (47), we tested the efficacy of camostat mesylate—a TMPRSS2 inhibitor (37)—and recombinant ACE2 (Figure 6). As shown in Figure 6B, rACE2 almost completely blocked infectivity by the pseudotyped virus, whereas TMPRSS2 had only a moderate impact. To determine whether the combination of factors released by infected EC might affect distant vessels—i.e., systemic effects—we collected effluent from the treated tissues and added this to EC transduced with an NF*κ*B promoter-mCherry reporter. NF*κ*B is a key pro-inflammatory transcription factor in EC. As shown in Figure 6C, we found strong induction of mCherry by supernatant from VMOs previously treated with virus and rAngII (and subsequently fed with fresh medium in the absence of virus or recombinant protein) that was apparent by 24 h of treatment. Interestingly, medium from VMOs treated with pseudotyped virus alone had no effect relative to control, indicating that systemic effects have a multi-factorial origin—loss of ACE2 in conjunction with Angiotensin II generation. Consistent with its effect on viral entry, rACE2 completely blocked the effects of pseudotyped virus effluent on NF*κ*B expression (Figure 6C). These data are therefore consistent with a mechanism whereby local infection of EC by SARS-CoV-2 could lead to systemic effects due to released cytokines and pro-coagulatory factors.

### 3.6 Later SARS-CoV-2 spike protein variants are also infectious in the VMO

**Figure 7:**
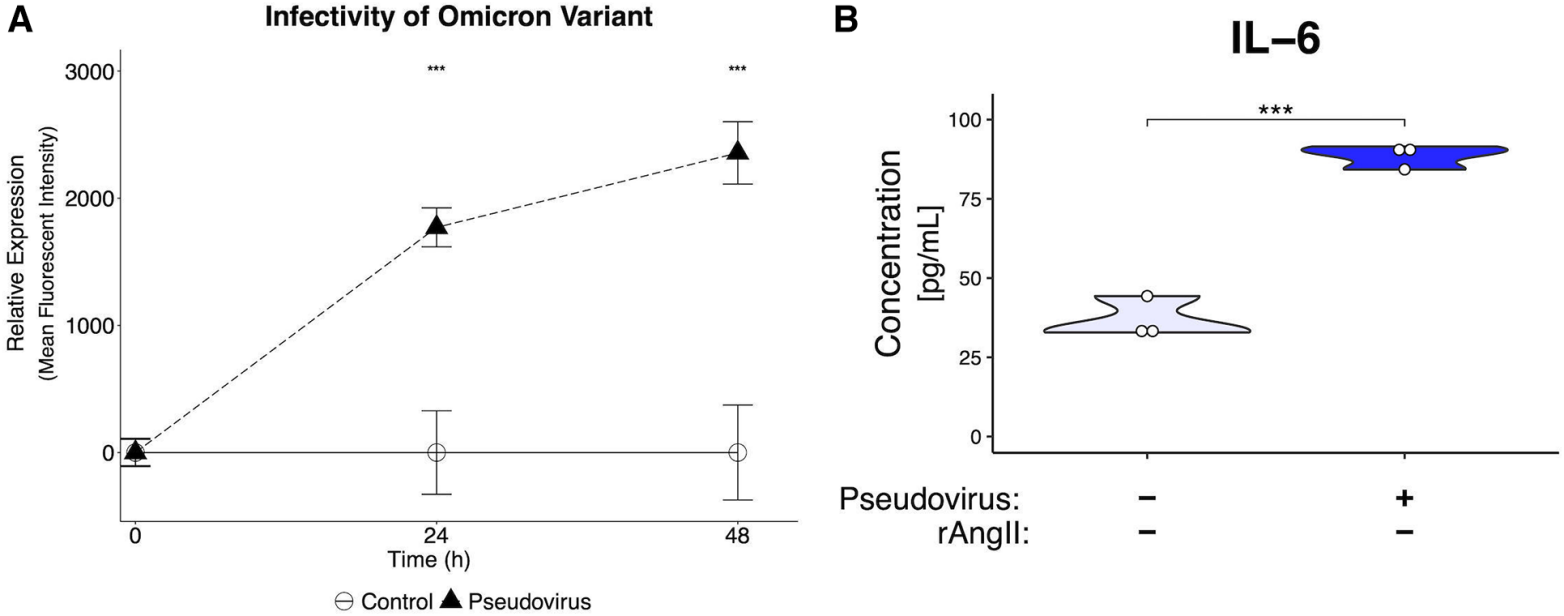
Generation of pseudotyped virus with omicron spike protein leads to infectivity and inflammatory response. (**A**) Mean fluorescent intensity of GFP signal for pseudotyped-virus with omicron variant spike protein. The virus can infect EC in the VMO. (**B**) ELISA of effluent from omicron-infected VMOs shows a significantly increased production of IL-6. ***<0.001.

To determine if the key findings generated using the pseudotyped virus carrying the original spike protein from 2019 could be replicated with the more a recent variant, a pseudotyped virus with the same backbone used for the 2019 variant was generated with the omicron BA.2 variant spike protein (23). The omicron pseudotyped virus was able to infect the VMO (Figure 7A), leading to strongly increased GFP expression. Importantly, effluent from infected VMOs had an increase in IL-6 cytokine levels (Figure 7B), consistent with our earlier results. Taken together, these data demonstrate the utility of the VMO for studying multiple SARS-CoV-2 variants.

## 4 Discussion

Leveraging the unique benefits that the VMO provides, we analyzed COVID-19 infection and the subsequent inflammatory response in a vascularized, fully human, and physiologically relevant system. Real-time fluorescent imaging allowed for direct quantification of the rate of viral entry within the endothelium, demonstrating the pivotal role of ACE2 in viral infection under shear conditions.

While previous work has relied on animal models or static monolayer studies to provide insight into the mechanisms that drive hypercytokinemia and tissue damage (48, 49), we show in this work the importance of flow in inducing the mRNA expression of ACE2, the SARS-CoV2 receptor. The internalization of ACE2 during viral entry removes the enzyme responsible for limiting the spread and duration of Angiotensin II effects. This loss of ACE2 results in prolonged pro-inflammatory signaling through AGTR1 and a loss of the negative, anti-inflammatory signal usually mediated by binding of the Angiotensin II breakdown product Ang_1–7_ to MasR.

Using the VMO allowed us to quantify this inflammatory response with various metrics, including immediate tissue changes in vessel morphometry and cytokine production, and effects of cytokine release from the infected cells on downstream, non-infected cells. The induction of NF*κ*Β activity in these non-infected cells is significant as this transcription factor is the key mediator of endothelial inflammatory responses, driving the expression of both cytokines and leukocyte adhesion molecules. Thus, the endothelium can be seen as an initiator and amplifier of the hypercytokinemia that proved so devastating to patients in the early stage of the pandemic. In addition to cytokine release, including upregulation of the pro-thrombotic cytokine IL-6, we show that viral infection also triggered the release of Factor VIII, a component of the intrinsic coagulation pathway. Thus, infected EC could contribute to hyper-coagulation in two ways, by direct release of Factor VIII and by release of IL-6, which can drive expression of other pro-thrombotic factors, including Tissue Factor (50), the initiator of clotting through the extrinsic pathway.

While using primary lung endothelial cells might enhance the findings of this study, we have found that commercially available primary EC from specific tissues mostly perform very poorly in vasculogenesis and angiogenesis assays, likely due to over-expansion prior to shipping. Indeed, we find that cells at passages below −5 work best in our VMO platform. That said, previous work from our group has shown that incorporating stromal cells from various sources, such as the lung or heart, leads to transcriptomic changes in the ECFC-EC, and that these model systems show similarity with *in vivo* data (51), suggesting that the stroma can influence EC phenotype. Additionally, unpublished data suggest that developing VMOs with NHLF upregulates lung marker expression on the EC, such as ACE, which a published study showed was only expressed at high and uniform levels in lung EC *in vivo*, with some organ vasculature being completely devoid of expression (52). Therefore, while primary lung endothelial cells may improve the translational impact of these findings, ECFC-EC are better suited for this work due to their vasculogenic and angiogenic potential, as well as plasticity in taking on tissue-specific phenotypes.

While COVID-19 mortality rates have been far lower since the introduction of mRNA vaccines (53), there is still a pressing need to better understand both the acute infection stage and the long-term effects of the disease following recovery. Currently, it is theorized that the profound impact of long-term COVID-19 is due to the dysregulation of thrombotic pathways leading to disseminated intravascular coagulation (54, 55). The VMO, along with other microphysiological systems, provides a unique avenue to study thrombosis and coagulation in a real-time system. Importantly, we have also demonstrated the utility of our platform for studying the impact of potential therapeutics such as rACE2 and camostat mesylate on reducing viral entry. In summary, we have demonstrated through the use of a sophisticated vascularized microphysiological system platform the critical role of flow in the induction of ACE2, and furthermore, have provided clear evidence that the vasculature could play a central role in the initiation and amplification of hypercytokinemia, a major driver of patient death in the early stages of the pandemic.

## Data Availability

The raw data supporting the conclusions of this article will be made available by the authors, without undue reservation.
