# Supplementary material for: SARS-CoV-2 infection of endothelial cells, dependent on flow-induced ACE2 expression, drives hypercytokinemia in a vascularized microphysiological system
Source: Front Cardiovasc Med. 2024 Mar 21;11:1360364. doi: 10.3389/fcvm.2024.1360364 (PMC10991679; doi:10.3389/fcvm.2024.1360364)
Supplement: Supplementary file 1 [file Table1.pdf]

Supplementary Material

1 Supplementary Figures

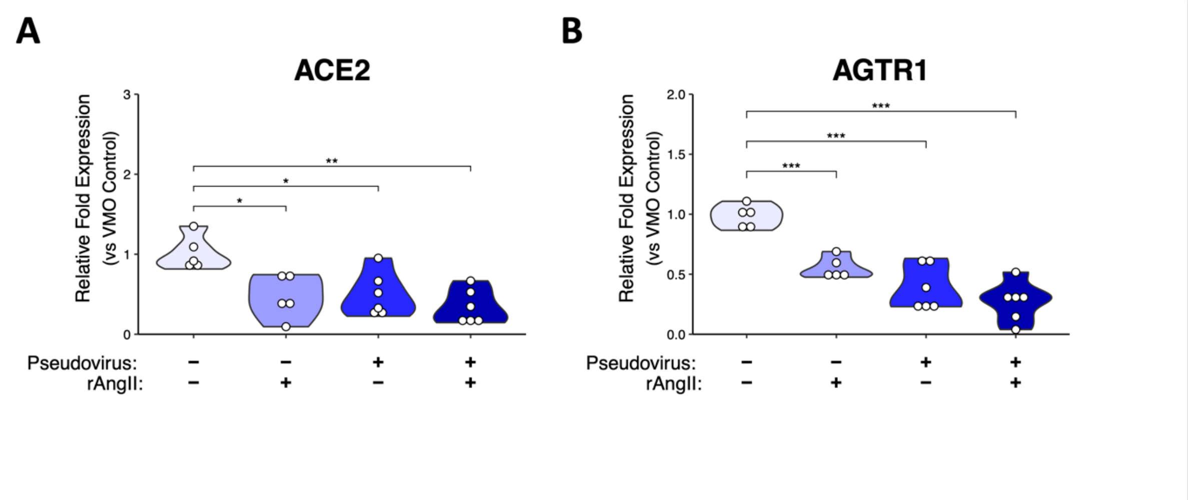

Supplemental Figure 1. Modeled SARS-CoV-2 infection alters VMO transcriptomic profiles. qPCR analysis of **(A)** ACE2 and **(B)** AGTR1 for perfused vascularized micro-organ (VMO) that have been treated with SARS-CoV-2 pseudotyped virus (pseudovirus), recombinant angiotensin II (rAngII) or both for 48 hours. * < 0.05, ** < 0.01, *** < 0.001.
